# Socio-economic and demographic factors associated with never having tested for HIV among sexually active men across the four administrative regions of Uganda

**DOI:** 10.1186/s12889-021-12384-2

**Published:** 2021-12-19

**Authors:** Otim Jude, Otim Nelson, Igeme Katagwa

**Affiliations:** Kyambogo University, Kampala, Uganda

**Keywords:** Never tested, sexually active men, HIV, regional, Uganda

## Abstract

**Background:**

HIV testing among men is paramount in the prevention, diagnosis, and treatment of HIV. There is limited literature in understanding the socio-economic and demographic factors associated with never having tested for HIV among sexually active men aged 15 – 54 across the four administrative regions of Uganda. The purpose of this study is to investigate the socio-economic and demographic factors associated with never having tested for HIV among sexually active men aged 15 – 54 across the four administrative regions in the country.

**Methods:**

The study used a cross-sectional research design to examine factors associated with never having tested for HIV among 4,168 sexually active men (15 – 54 years) across four administrative regions of Uganda using data from 2016 Uganda Demographic and Health Survey (UDHS). Frequency distributions, Pearson chi-square tests, and multivariable logistic regression were used to establish the association between never having tested for HIV among sexually active men (15 – 54 years) and selected independent variables across regions.

**Results:**

About 20% of sexually active men (15 – 54 years) never tested for HIV across regions of the country. The major correlates amidst variability of never testing for HIV among sexually active men across regions were; educational level and marital status. Age, religious status, wealth quintile, worked in the last 12 months, circumcised, and one sexual partner in the last 12 months were only correlates of never having tested for HIV among respondents in particular regions of the country.

**Conclusion:**

Findings in the study suggest promotion of male education, and suggest further investigation into the relationship between HIV non-testing among sexually active men (15 – 54 years) and being married across regions of the country. The study also proposes appreciation of regional differences in the outcome of HIV non-testing and suggests that efforts be focused on addressing regional differences in order to attain high HIV testing among sexually active men (15 – 54 years) across regions of Uganda, and thus reduce HIV related morbidity and mortality.

## Background

Human Immunodeficiency Virus (HIV) remains a major global health concern that weakens body immunity, and causes many HIV related deaths [1]. HIV/AIDS is a main cause of morbidity and mortality in sub-Saharan Africa [2, 3]. Regardless of rise in uptake of Anti-Retroviral Therapy (ART) in the mid-2000s and the consequent decline in mortality within sub-Saharan Africa, eastern and southern Africa is the hardest hit region by HIV, with more than 54% (20.7 million) of the total number of people living with the disease, including 7.3 million HIV positive males [4, 5]. Men and women in the region are prone to contracting HIV, however men are less likely to test for HIV, more likely to be diagnosed with advanced stages of the disease and more likely to be associated with HIV related deaths because of their poorer testing uptake and treatment [6–12]. HIV testing is a crucial approach in reducing HIV associated morbidity and mortality outcomes [13–15]. According to the World Health Organization (WHO) and Center for Disease Control (CDC), HIV testing is an essential pathway to prevention and management of HIV [16, 17]. It equips individuals with the knowledge to evade infection, as well as a prerequisite to initiate ART [18]. Notably, differences in HIV prevalence and proportion of never having tested for HIV among men in sub-Saharan Africa exist, even within countries [12, 19].

Uganda has four administrative regions which include; eastern, western, northern and central regions [20]. These regions differ in relation to social context, poverty share, and health infrastructure. For example; northern region is the least economically and socially developed region of Uganda, central and western regions have better health infrastructure compared to other regions [21, 22]. Historically, central region was the path of social services into the country due to earlier contact with western influences [23]. Consequently, most social services first became rooted in the area before spreading out to other regions of the country [23–25]. Central region also hosts the national capital, Kampala, and is the most urbanized [26, 27]. Systems of education, health, and communication are better in central region than in other regions [21]. In contrast, northern region has gone through over two decades of insurgency [28, 29] which has adversely affected the ability to manage health challenges [30]. Literature shows that social context, poverty, and health infrastructure have an impact on never having tested for HIV among men [31–36].

In Uganda, the proportion of men who have never tested for HIV is high (27%), yet over 80% of all men across the country have knowledge on where to obtain HIV testing services [37, 38] amidst availability of free HIV testing [39, 40]. Men’s HIV testing coverage in the country is a major hindrance to HIV decline [32, 41], and achievement of the global commitment to reduce and end the epidemic by 2030 [42, 43]. Consequently, emphasis ought to be placed in understanding the challenges men face in testing for HIV. Studies on drivers of never having tested for HIV among men in Uganda indicate among others; fear of being tested, older age, fear of knowing HIV status, absence of testing interest, men’s view of clinics as places for females, culture, facilities, fear of testing-related gossip, distrust of HIV testing methods, peer and economic influence [10, 32, 44–56], without examining determinants of never having tested for HIV among sexually active men (15 -54 years) across regions of Uganda. In Uganda, regional differences in never having tested for HIV among men exist, for instance; highest in Karamoja region (60%), trailed by Bukedi region (42%) and lowest in Greater Kampala (14%), betwixt availability of HIV testing services [20, 57]. These variations in never having tested for HIV among men across regions of the country signify a possibility of regional differences in determinants of never having tested for HIV.

## Methods

### Study design, data sources and population

**Fig 1:**
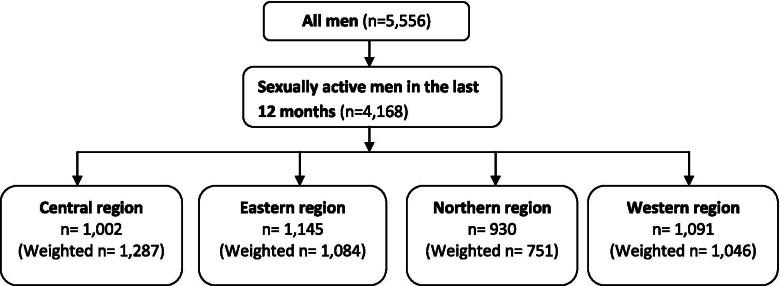
Origin of the sample used for the study

**Fig. 2:**
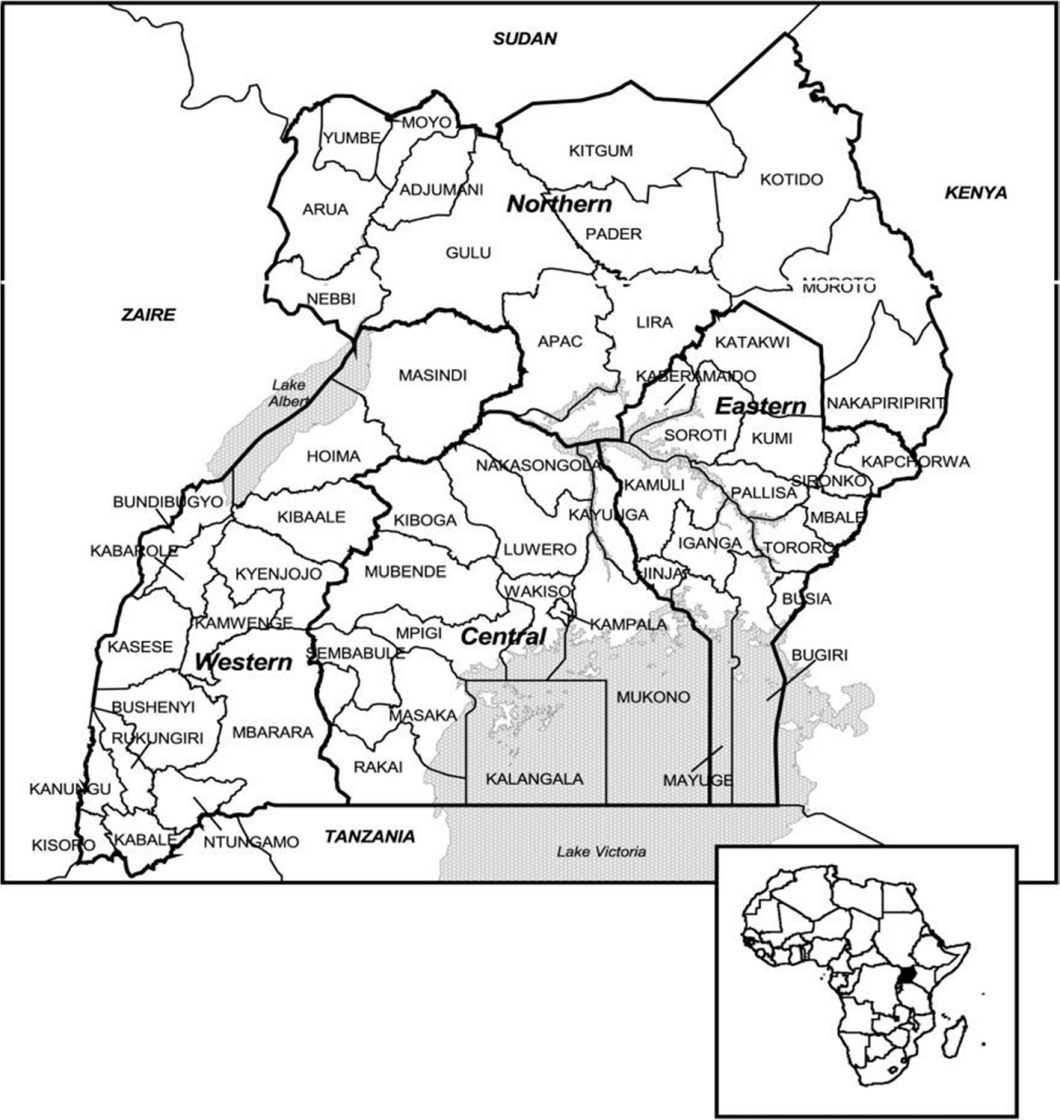
Regions of Uganda and their respective districts *(Source: UBOS and ORC Macro, 2001)*

The present study used a cross-sectional research design. The 2016 Uganda Demographic and Health Survey (UDHS) was the premise of the present study. The 2016 UDHS was a national cross-sectional survey that captured the socio-economic, and demographic related issues concerning men aged 15 - 54. A two-stage cluster sampling was used in acquiring a representative sample of 5,336 men (15 – 54 years). Selection of clusters was undertaken in the first stage, whilst selection of the households was handled in the second stage of cluster sampling. The present study only selected 4,168 men who reported to have had sexual intercourse in the last 12 months. Notably, sexual activity is a key aspect through which HIV is transmitted [58]. Out of the 15 regions that were captured in the 2016 UDHS, the present study grouped the regions into four (4) administrative regions of Uganda; central, eastern, northern and western region (10). Study approval by the Institutional Review Board (IRB) was not applicable, since the research utilized secondary data. While using STATA 13.1, this study weighted and adjusted the 2016 UDHS dataset for non-responses in order to ascertain sample representativeness. These weighted counts can and usually will be non-integers. In order to address multicollinearity, a variance inflation factor (VIF) test was undertaken [59]. In the test, we examined our data and omitted extremely correlated covariates from the models. We as well did a multiple regression analysis including religion for sensitivity analysis. For additional information on the sampling procedures, exhaustive clarification can be obtained elsewhere [38]. Figure 1 is a flow chart that demonstrates how the study sample was obtained. Figure 2 is a map of Uganda showing the four administrative regions and their respective districts [60].

## Study variables

### Outcome variable

The outcome variable in this present study was self-reported lifetime history of HIV testing among men aged 15 – 54. The 2016 UDHS men’s questionnaire, asked respondents whether they have ever tested for HIV (‘have you ever tested for HIV?’). From the question, a binary outcome variable was produced, and coded: ‘1’ never tested for HIV and ‘0’ ever tested for HIV (yes =1, no = 0).

### Explanatory variable

This study selected explanatory variables of HIV non-tested among sexually active men (15 – 54 years) based on literature review [12, 61–65] and the range of the 2016 UDHS variables [38]. The chosen explanatory variables are; age (grouped into four distinct categories; 15 – 24, 25 – 34, 35 – 44, and 45 – 54), education status (no education, primary, secondary, and higher), residence (categorized as: urban, and rural), religious status (Catholic, Anglican, Muslim, Pentecostal, and others), wealth quintile (poorest, poorer, middle, richer, and richest), worked in the last 12 months (yes/ no), ever given gifts or other goods to have sex in the last 12 months (no/yes), marital status (never married, married, widowed, and separated), circumcised (no/yes), and number of sexual partners, excluding spouse in the last 12 months (No sexual partner, 1 sexual partner, 2 and above sexual partners).

### Ethical considerations

Demographic and Health Survey (DHS) dataset can freely be accessed by the public upon submission of a formal request. Therefore, as a pre-requisite, authors submitted a proposal to DHS Program/ICF International which later approved the download and use of the 2016 UDHS dataset. Further information on ethical concerns is elucidated elsewhere [38].

### Statistical analysis

This study utilized STATA 13.1 in executing the three stages of statistical analyses. In the first stage, characteristics of study respondents across regions of the country were presented using descriptive statistics. In the second stage of the analysis, associations between each explanatory variable and outcome variable across regions of the country were evaluated with the Pearson chi-square test (*x*^2^). In the third stage of analysis, the study used multivariable logistic regression to model the associations between lifetime HIV testing history and the explanatory variables among sexually active men, across regions. At the second stage of analysis (bivariate), some variables were not statistically significant across regions (residence, religious status, wealth quintile, worked in the last 12 months, ever given gifts or other goods to have sex in the last 12 months, and number of sexual partners in the last 12 months), however, they were retained for further analysis due to their published associations with HIV testing history [12, 64–68]. Odds ratios (ORs) and 95% confidence intervals were measured and adopted at the multivariable logistic regression. Further, p-values less than 0.001 reported very strong relationship between variables, p-values less than 0.01 showed a strong relationship between variables, and p-values less than 0.05 demonstrated moderate relationship between variables [69].

## Results

### Descriptive findings

Results in Table 1 show that 19.8% of respondents in Uganda have never tested for HIV, with variations across regions; eastern region (23.6%) had the largest proportion of respondents who have never tested for HIV, unlike northern region (15.1%). In Table 1, central, eastern, northern and western regions, had the largest proportion of respondents with primary education (44.8%, 54.8%, 56.8%, and 60.3%, respectively). Majority of the respondents in the country were catholics (39.9%) and anglicans (34.5%), with the largest proportion of respondents in central (39.1%) and northern regions (61.5%), whereas in eastern and western regions, the largest proportion of respondents were anglicans (41.8% and 42.6%, respectively). Results (Table 1) show that majority of the respondents in the country were in the richest wealth quintile (24.6%); however, differences across regions indicated that central region (41.3%) had the largest proportion of respondents in the richest wealth quintile category, while northern region had the largest proportion in the poorest wealth quintile (45.1%). Across the four regions, over three-quarters had ever been married; the largest proportion married at least once were in northern region (82.7%) (Table 1). Results in Table 1 show that 53.8% of the respondents reported not have been circumcised in the entire country, with the largest proportion in eastern (57.1%) and central region (52.5%), and lowest in northern region (24.1%). Besides, results in Table 1 also indicate that majority of respondents in the country had no sexual partner in the last 12 months (62.3%), followed by respondents with 1 sexual partner in the last 12 months (29.4%), and least are those who had 2 and more sexual partners in the last 12 months (8.3%). Notable, regional variations indicate that the highest proportion of the respondents with no sexual partner, 1 sexual partner, and 2 and more sexual partners in the last 12 months were in northern (66.8%), central (34.4%), and central region (11.0), respectively.

**Table 1:** Lifetime HIV testing history, socio-economic and demographic characteristics of sexually active men (15 – 54 years) across regions of Uganda

Characteristics	Regions								Uganda (n=4,168)	
	Central (n=1,002)		Eastern (n=1,145)		Northern (n=930)		Western (n=1,091)			
	**n**	**%**	**n**	**%**	**n**	**%**	**n**	**%**	**n**	**%**
**HIV testing status**										
Never tested	187	18.7	271	23.6	140	15.1	223	20.4	823	19.8
Ever tested	815	81.3	875	76.4	790	84.9	868	79.6	3345	80.2
**Age**										
15 – 24	292	29.2	348	30.4	250	26.9	321	29.4	1214	29.1
25 – 34	361	36.0	358	31.3	331	35.5	360	33.0	1415	33.9
35 – 44	232	23.2	281	24.6	200	21.5	249	22.8	966	23.2
45 – 54	116	11.6	158	13.8	149	16.0	161	14.8	574	13.8
**Education level**										
No education	47	4.6	46	4.0	38	4.0	59	5.4	191	4.6
Primary	449	44.8	628	54.8	528	56.8	658	60.3	2228	53.5
Secondary	309	30.9	353	30.8	205	22.1	268	24.6	1154	27.7
Higher	197	19.7	118	10.3	159	17.1	106	9.7	595	14.3
**Residence**										
Urban	460	45.9	174	15.2	120	12.9	239	21.9	1081	25.9
Rural	542	54.1	971	84.8	810	87.1	852	78.1	3087	74.1
**Religious status**										
Catholic	391	39.1	330.	25.5	573	61.5	440	40.3	1664	39.9
Anglican	274	27.4	478	41.8	231	24.8	464	42.6	1437	34.5
Muslim	202	20.1	221	19.3	55	5.9	62	5.7	572	13.7
Pentecostal	113	11.2	134	11.7	60	6.5	65	5.9	382	9.2
Others	22	2.2	20	1.7	12	1.3	60	5.5	114	2.7
**Wealth quintile**										
Poorest	27	2.7	241	21.0	420	45.1	82	7.5	681	16.3
Poorer	71	7.1	281	24.5	218	23.4	205	18.8	729	17.5
Middle	154	15.3	258	22.6	97	10.5	314	28.7	821	19.7
Richer	261	26.1	216	18.9	109	11.7	296	27.1	912	21.9
Richest	489	48.8	149	13.0	86	9.3	195	17.8	1025	24.6
**Worked in the last 12 months**										
No	21	2.1	17	1.5	13	1.4	34	3.1	87	2.1
Yes	981	97.9	1128	98.5	917	98.6	1057	96.9	4081	97.9
**Marital status**										
Never married	307	30.6	237	20.7	173	18.6	246	22.6	994	23.8
Ever married	695	69.4	908	79.3	757	81.4	845	77.4	3174	76.2
**Ever given gifts or other goods to have sex in the last 12 months**										
No	875	87.3	1080	94.3	917	98.6	1006	92.2	3851	92.4
Yes	127	12.7	65	5.7	14	1.4	85	7.8	317	7.6
**Circumcised**										
No	476	47.5	492	42.9	706	75.9	622	57.0	2243	53.8
Yes	526	52.5	653	57.1	224	24.1	469	43.0	1925	46.2
**Number of sexual partners in the last 12 months**										
No sexual partner	548	54.6	791	69.1	621	66.8	669	61.3	2595	62.3
1 sexual partner	345	34.4	293	25.6	254	27.3	313	28.7	1226	29.4
2 & above sexual partners	110	11.0	61	5.3	54	5.8	109	10.0	347	8.3

*Appropriate weights were used for the analyses.*

### An assessment of variations in socio-economic and demographic factors by HIV testing status among sexually active men (15 – 54 years) across regions of Uganda

In Table 2, results show an assessment of socio-economic and demographic factors by HIV testing status among sexually active men (15 – 54 years) across regions of the country. Table 2 shows results at bivariate level of analysis; herein, Pearson’s Chi-square tests (*x*^2^) were performed, specifically highlighting the relationship between respondents’ socio-economic and demographic factors and HIV testing status across regions. In Table 2, results show that age, education level, residence, wealth quintile, worked in the last 12 months, and marital status were significantly associated with HIV testing status among sexually active men (15 – 54 years) in Uganda, with variations across region; age, educational level, and marital status were the only variables that were significantly associated with HIV testing status among sexually active men (15 – 54 years) across regions (p-value < 0.001). Nevertheless, this study adopted all the variables at the multivariable level of analysis because of their importance in explaining HIV non-testing among sexually active men [12, 61–65].

**Table 2:** Distribution of socio-economic and demographic factors by HIV testing status among sexually active men aged 15 – 54 across regions

Characteristics	Regions								Uganda	
	Central		Eastern		Northern		Western			
	Ever tested n (%)	Never tested n (%)	Ever tested n (%)	Never tested n (%)	Ever tested n (%)	Never tested n (%)	Ever tested n (%)	Never tested n (%)	Ever tested n (%)	Never tested n (%)
**Age**										
15 – 24	217 (74.3)	75 (25.7)	237 (68.2)	110 (31.8)	191 (76.2)	60 (23.8)	217 (67.6)	104 (32.4)	865 (71.3)	349 (28.7)
25 – 34	314 (87.0)	47 (13.0)	282 (78.7)	76 (21.3)	307 (93.0)	23 (7.0)	316 (87.8)	44 (12.2)	1222 (86.3)	193 (14.7)
35 – 44	187 (80.6)	45 (19.5)	226 (80.4)	55 (19.6)	178 (88.8)	23 (11.2)	213 (85.3)	37 (14.7)	802 (83.1)	164 (16.9)
45 – 54	97 (83.0)	20 (17.0)	130 (82.1)	28 (17.9)	114 (76.5)	35 (23.5)	123 (76.2)	38 (23.8)	457 (79.6)	117 (20.4)
	**χ**^**2**^**= 3.5524, p=0.0142**		**χ**^**2**^**= 4.2277, p≤0.0064**		**χ**^**2**^**= 11.8350, p≤0.001**		**χ**^**2**^**= 13.4259, p≤0.001**		**χ**^**2**^**= 21.4522, p≤0.001**	
**Education level**										
No education	25 (53.9)	22 (46.1)	29 (61.9))	18 (38.1)	13 (33.3)	25 (66.7)	43 (71.5)	17 (28.5)	110 (57.7)	81(42.3)
Primary	338 (75.3)	111 (24.7)	441 (70.3)	187 (29.7)	430 (81.3)	99 (18.7)	507 (77.3)	150 (22.7)	1686 (75.7)	543 (24.4)
Secondary	261 (84.3)	48 (15.7)	294 (83.5)	58 (16.5)	195 (94.8)	11 (5.2)	219 (81.8)	49 (18.2)	981 (85.0)	173 (15.0)
Higher	191 (96.9)	6 (3.1)	110 (93.3)	8 (6.7)	153 (96.6)	5 (3.4)	99 (93.0)	7 (7.0)	568 (95.5)	27 (4.5)
	**χ**^**2**^**= 16.6242, p≤0.001**		**χ**^**2**^**= 11.0568, p≤0.001**		**χ**^**2**^**= 39.2516, p≤0.001**		**χ**^**2**^**= 5.1079, p=0.0018**		**χ**^**2**^**= 46.9833, p≤0.001**	
**Residence**										
Urban	404 (88.0)	55 (12.0)	141 (80.9)	33 (19.1)	107 (89.2)	13 (10.8)	191 (80.0)	48 (20.0)	923 (85.3)	159 (14.7)
Rural	410 (75.7)	132 (24.3)	734 (75.6)	237 (24.4)	683 (84.3)	127 (15.7)	677 (79.5)	175 (20.5)	2423 (78.5)	665 (21.5)
	**χ**^**2**^**= 15.5287, p≤0.001**		χ^2^= 1.0799, p=0.2991		χ^2^= 1.9159, p=0.1668		χ^2^= 0.0210, p=0.8848		**χ**^**2**^**= 12.5572, p=0.004**	
**Religious status**										
Catholics	311 (79.6)	80 (20.4)	225 (76.8)	68 (23.2)	486 (84.9)	86 (15.1)	337 (76.6)	103 (23.4)	1329 (79.9)	335 (20.1)
Anglican	230 (83.9)	44 (16.1)	371 (77.5)	108 (22.5)	200 (86.7)	31 (13.3)	375 (80.7)	90 (19.3)	1167 (81.2)	270 (18.8)
Muslim	153 (76.2)	48 (23.8)	153 (69.4)	68 (30.6)	44 (81.0)	10 (19.0)	53 (86.2)	9 (13.8)	429 (75.1)	142 (24.9)
Pentecostal	100 (88.3)	13 (11.7)	111 (83.0)	23 (17.0)	55 (90.6)	6 (9.4)	55 (85.2)	10 (14.8)	330 (86.3)	52 (13.7)
Others	20 (93.3)	2 (6.7)	15 (76.4)	5 (23.6)	5 (40.2)	7 (59.8)	48 (79.8)	12 (20.2)	90 (79.2)	24 (20.8)
	χ^2^= 2.2809, p=0.0639		χ^2^=1.6889, p=0.1516		**χ**^**2**^**= 4.1604, p=0.037**		χ^2^= 1.0481, p=0.3765		χ^2^= 3.3290, p=0.0105	
**Wealth quintile**										
Poorest	19 (69.5)	8 (30.5)	178 (73.8)	63 (26.3)	343 (81.7)	77 (18.3)	50 (60.7)	32 (39.3)	517 (76.0)	163 (24.0)
Poorer	44 (62.4)	27 (37.6)	210 (74.9)	70 (25.1)	188 (86.3)	30 (13.7)	158 (76.9)	47 (23.1)	559 (76.7)	170 (23.3)
Middle	109 (71.0)	44 (29.0)	184 (71.1)	75 (28.9)	81 (83.1)	17 (16.9)	254 (81.0)	60 (19.0)	623 (75.8)	199 (24.2)
Richer	195 (74.5)	67 (25.5)	179 (82.9)	37 (17.1)	97 (88.6)	13 (11.4)	236 (79.7)	60 (20.3)	724 (79.4)	188 (20.6)
Richest	448 (91.6)	41 (8.4)	124 (83.1)	25 (16.9)	81 (94.6)	5 (5.4)	171 (87.9)	24 (12.1)	923 (90.0)	103 (10.0)
	**χ**^**2**^**= 12.0630, p≤0.001**		χ^2^= 2.0527, p=0.0935		χ^2^= 2.1795, p=0.0725		**χ**^**2**^**= 5.9923, p≤0.001**		**χ**^**2**^**= 13.5046, p≤0.001**	
**Worked in the last 12 months**										
No	12 (57.0)	9 (43.0)	8 (49.0)	9 (51.0)	7 (55.8)	6 (44.2)	23 (67.3)	11 (32.7)	51 (59.2)	35 (40.8)
Yes	803 (81.9)	178 (18.1)	866 (76.8)	262 (23.2)	783 (85.4)	134 (14.6)	845 (80.0)	212 (20.0)	3294 (80.7)	788 (19.3)
	**χ**^**2**^**= 6.2645, p=0.0125**		**χ**^**2**^**= 6.0192, p=0.0144**		**χ**^**2**^**= 12.7655, p≤0.001**		χ^2^= 1.7484, p=0.1865		**χ**^**2**^**= 16.2038, p≤0.001**	
**Marital status**										
Never married	229 (74.8)	77 (25.2)	148 (62.5)	89 (37.5)	128 (74.0)	45 (26.0)	155 (63.0)	91 (37.0)	687 (69.1)	307 (30.9)
Ever married	586 (84.2)	110 (15.8)	727 (80.0)	182 (20.0)	662 (87.4)	95 (12.6)	713 (84.4)	132 (15.6)	2658 (83.7)	516 (16.3)
	**χ**^**2**^**= 6.5313, p=0.0108**		**χ**^**2**^**= 16.1246, p≤0.001**		**χ**^**2**^**=12.7302, p≤0.001**		**χ**^**2**^**= 34.7689, p≤0.001**		**χ**^**2**^**= 53.1542, p≤0.001**	
**Ever given gifts or other goods to have sex in the last 12 months**										
No	717 (82.0)	158 (18.0)	831 (76.9)	249 (23.1)	781 (85.2)	136 (14.8)	800 (79.5)	206 (20.5)	3105 (80.0)	746 (19.4)
Yes	98 (77.0)	29 (23.0)	43 (66.9)	22 (33.1)	9 (68.7)	4 (31.3)	69 (80.7)	16 (19.3)	240 (75.7)	77 (24.3)
	χ^2^= 1.4311, p=0.2320		χ^2^= 3.7218, p=0.0541		χ^2^= 2.7495, p=0.0977		χ^2^= 0.0388, p=0.8439		χ^2^= 3.2888, p=0.0702	
**Circumcised**										
No	369 (77.5)	107 (22.5)	379 (77.1)	113 (22.9)	606 (85.8)	100 (14.2)	473 (76.0)	149 (24.0)	1775 (79.1)	469 (20.9)
Yes	446 (84.8)	80 (15.2)	496 (75.9)	158 (24.1)	184 (82.2)	40 (17.8)	396 (84.3)	73 (15.7)	1570 (81.6)	354 (18.4)
	**χ**^**2**^**= 5.0290, p=0.0252**		χ^2^= 0.1328, p=0.7157		χ^2^=1.0156, p=0.3139		**χ**^**2**^**= 8.7932, p=0.0031**		χ^2^= 2.4351, p=0.1191	
**Number of sexual partners in the last 12 months**										
No sexual partner	448 (81.8)	99 (18.2)	625 (79.0)	166 (21.0)	536 (86.2)	86 (13.8)	548 (82.0)	120 (18.0)	2126 (81.9)	469 (18.1)
1 sexual partner	281 (81.5)	64 18.5)	205 (69.7)	89 (30.3)	212 (83.2)	43 (16.8)	247 (79.0)	66 (21.0)	962 (78.5)	264 (21.5)
2 & above sexual partners	86 (78.4)	24 (21.6)	45 (73.9)	16 (26.1)	43 (78.2)	12 (21.8)	73 (66.5)	37 (33.5)	257 (74.1)	90 (25.9)
	χ^2^= 0.2288, p=0.7738		**χ**^**2**^**= 4.0041, p=0.0192**		χ^2^= 1.3207, p=0.2672		**χ**^**2**^**= 5.9696, p=0.0029**		χ^2^= 5.3051, p=0.0054	

*Weighted proportions; ever tested (ever tested for HIV); never tested (never tested for HIV).*

### Socio-economic and demographic factors associated with never having tested for HIV among sexually active men aged (15 – 54 years) across regions in Uganda

Results in Table 3 reveal the socio-economic and demographic factors associated with never having tested for HIV among sexually active men aged 15 – 54 across regions in Uganda, premised on adjusted multivariable logistic regression. The results (Table 3) indicate that age, education level, religious status, wealth quintile, worked in the last 12 months, marital status, circumcised, and number of sexual partners in the last 12 months were factors associated with never having tested for HIV among sexually active men (15 – 54 years) in Uganda, with variations in impact of the variables across regions. For instance, educational level and marital status were the only factors associated with never having tested for HIV amongst respondents amidst variation in effect across all the regions. Age, religious status, wealth quintile, worked in the last 12 months, circumcised, and number of sexual partners in the last 12 months were only predictors of never having tested for HIV among respondents in particular regions.

**Table 3:** Multivariable logistic regression analysis on the net-impact of factors associated with never having tested for HIV among sexually active men (15 – 54 years) across regions

Characteristics	Regions								Uganda	
	Central		Eastern		Northern		Western			
	OR	95%CI	OR	95%CI	OR	95%CI	OR	95%CI	OR	95%CI
**Age**										
15 – 24†	1.00	.	1.00	.	1.00	.	1.00	.	1.00	.
25 – 34	0.82	0.44-1.53	1.00	0.63-1.61	0.47*	0.23-0.97	0.49*	0.28-0.87	0.72*	0.54-0.96
35 – 44	1.34	0.63-2.84	0.87	0.51-1.48	0.63	0.29-1.36	0.62	0.33-1.16	0.86	0.62-1.18
45 – 54	0.90	0.39-2.07	0.77	0.42-1.43	1.72	0.81-3.65	1.16	0.60-2.25	1.02	0.73-1.44
**Education level**										
No education†	1.00	.	1.00	.	1.00	.	1.00	.	1.00	.
Primary	0.32**	0.16-0.66	0.61	0.25-1.48	0.10***	0.04-0.24	0.69	0.36-1.32	0.38***	0.26-0.54
Secondary	0.19***	0.08-0.44	0.26**	0.10-0.66	0.02***	0.01-0.06	0.45*	0.22-0.95	0.19***	0.12-0.28
Higher	0.06***	0.02-0.23	0.12**	0.04-0.41	0.01***	0.003-0.05	0.26*	0.09-0.77	0.07***	0.04-0.13
**Residence**										
Rural†	1.00	.	1.00	.	1.00	.	1.00	.	1.00	.
Urban	1.31	0.72-2.37	0.99	0.55-1.79	1.32	0.61-2.86	1.28	0.83-1.97	1.15	0.88-1.54
**Religious status**										
Catholics†	1.00	.	1.00	.	1.00	.	1.00	.	1.00	.
Anglican	0.87	0.51-1.49	0.91	0.61-1.35	0.99	0.58-1.69	0.95	0.65-1.37	0.97	0.78-1.21
Muslim	2.22*	1.17-4.21	1.46	0.88-2.43	1.06	0.39-2.88	0.93	0.35-2.48	1.73**	1.25-2.39
Pentecostal	0.56	0.26-1.19	0.78	0.41-1.47	0.91	0.33-2.52	0.49	0.24-1.03	0.70	0.48-1.02
Others	0.45	0.09-2.27	0.93	0.23-3.77	2.70	0.37-19.47	0.80	0.37-1.71	0.91	0.55-1.53
**Wealth quintile**										
Poorest†	1.00	.	1.00	.	1.00	.	1.00	.	1.00	.
Poorer	1.22	0.37-4.02	1.06	0.67-1.67	1.01	0.56-1.78	0.39**	0.20-0.77	1.05	0.79-1.39
Middle	0.89	0.27-2.90	1.19	0.75-1.89	1.19	0.57-2.49	0.28***	0.15-0.53	0.99	0.75-1.32
Richer	0.87	0.28-2.75	0.72	0.39-1.30	0.85	0.40-1.83	0.27***	0.14-0.52	0.90	0.67-1.21
Richest	0.22*	0.06-0.75	0.89	0.43-1.85	0.49	0.11-2.14	0.17***	0.08-0.37	0.42***	0.29-0.62
**Worked in the last 12 months**										
Yes†	1.00	.	1.00	.	1.00	.	1.00	.	1.00	.
No	2.65	0.79-8.94	2.22	0.84-5.89	3.15*	1.18-8.42	1.21	0.45-3.29	2.09**	1.22-3.60
**Marital status**										
Ever married†	1.00	.	1.00	.	1.00	.	1.00	.	1.00	.
Never married	4.52***	2.12-9.63	2.50**	1.42-4.38	4.50***	2.01-10.03	3.69***	2.00-6.79	3.46***	2.46-4.89
**Ever given gifts or other goods to have sex in the last 12 months**										
No†	1.00		1.00		1.00		1.00		1.00	
Yes	0.98	0.53-1.78	1.12	0.60-2.07	3.25	0.72-14.76	0.69	0.35-1.36	0.99	0.70-1.41
**Circumcised**										
No†	1.00	.	1.00	.	1.00	.	1.00	.	1.00	.
Yes	0.58*	0.35-0.98	0.99	0.68-1.46	1.25	0.69-2.26	0.51**	0.35-0.76	0.81*	0.65-1.00
**Number of sexual partners in the last 12 months**										
No sexual partner†	1.00	.	1.00	.	1.00	.	1.00	.	1.00	.
1 sexual partner	0.55*	0.31-0.98	1.10	0.69-1.76	0.81	0.42-1.58	0.73	0.44-1.19	0.75*	0.57-0.98
2 & above sexual partners	0.53	0.23-1.22	0.70	0.32-1.54	0.70	0.27-1.83	1.28	0.68-2.42	0.81	0.54-1.21

**Abbreviations:** ^**†**^Reference category. Significant *(P < 0.05)*, *P < 0.05 (Moderate), **P < 0.01 (Strong) ***P < 0.001 (very strong), ***OR*** Odds ratio, ***CI*** Confidence Interval

Notably, in Uganda (Table 3), amidst very strong significance (P < 0.001), increase in educational level was associated with reduced odds of never to have tested for HIV among respondents amid variations across regions; a very strong significance of education level was reported only in northern (primary, secondary, and higher vs no education), and central region (secondary and higher vs no education). Results in Table 3 show that, with a very strong significance, never married respondents were 3.46 times (OR= 3.46; 95% CI = 2.46-4.86) more likely to have never tested for HIV in Uganda amidst regional variations; never married respondents were 4.52 times with a very strong significance (OR = 4.52; 95% CI = 2.12-9.63), 2.50 times with a strong significance (OR = 2.50; CI = 1.42-4.38), 4.50 times with a very strong significance (OR = 4.50; CI = 2.01-10.03), and 3.69 times with a very strong significance (OR = 3.69; CI = 2.00-6.79), in central, eastern, northern and western region respectively, more likely to have never tested for HIV compared with their counterparts who have ever married.

Results in Table 3 show that amidst moderate significance (P < 0.05), respondents aged 25 – 34 were less likely (OR = 0.72; CI = 0.54-0.96) to have ever tested for HIV compared to the respondents aged 15 – 24 in Uganda. However, only respondents aged 25 – 34 in northern and western region demonstrated less likelihoods of never having tested for HIV amidst moderate significance (OR = 0.47; CI = 0.23-0.97, and OR = 0.49; CI = 0.28-0.87 respectively) compared with their counterparts aged 15 – 24.

In addition, results (Table 3) indicate that muslim respondents were 1.78 times amidst a strong significance (OR = 1.78; CI = 1.25-2.39), more likely never to have tested for HIV compared with the catholics in Uganda. However, with scrutiny of effect across regions, Table 3 shows that only muslim respondents in central region were 2.21 times amidst moderate significance (OR = 2.21; CI = 1.16-4.21), more likely to have never tested for HIV compared with their counterparts the catholics.

Results in Table 3 reveal that respondents in the richest wealth quintile were 0.42 times, amidst very strong significance (OR = 0.42; CI = 0.29-0.62), less likely never to have tested for HIV compared with the respondents in the poorest wealth quintile in Uganda. However, analysis on wealth quintile across regions in Table 3 demonstrated differences in effect across regions; in western region amidst strong (poorer) and very strong associations (middle, richer, and richest), an increase in wealth quintile among sexually active men (15-54 years) was associated with reduced odds of never to have tested for HIV amongst respondents. In addition, with a moderate association in central region, sexually active men in the richest wealth quintile had reduced odds of never to have tested for HIV (OR = 0.22; CI = 0.06-0.75) compared with the respondents in the poorest wealth quintile.

Besides, results in Table 3 demonstrate that, with a strong significance, respondents who did not work in the last 12 months were more likely (OR = 2.09; CI = 1.22-3.60) to have never tested for HIV compared with their counterparts who worked in the last 12 months in Uganda. However, analysis across regions (Table 3) shows that, with moderate significance, respondents in northern region who did not work in the last 12 months, had increased odds of never to have tested for HIV (OR = 3.15; CI = 1.18-8.42), compared with the respondents who worked in the last 12 months.

Further, results in Table 3 reveal that respondents who reported to have been circumcised in Uganda were less likely (OR = 0.81; CI = 0.65-1.00) to have never tested for HIV compared with their counterparts who reported not to be circumcised, amid strong significance. However, analysis across regions only indicated variation in association (moderate and strong in central and western region, respectively); where sexually active circumcised men in central and western regions were 0.58 times (OR = 0.58; CI = 0.35-0.98) and 0.51 times (OR = 0.51; CI = 0.35-0.76) respectively, less likely to have never tested for HIV compared with their counterparts the uncircumcised.

Results in Table 3 demonstrate that respondents who reported to have had 1 sexual partner amid moderate significance were less likely (OR = 0.75; CI = 0.57-0.98) to have never tested for HIV compared with respondents who reported to have had no sexual partner in the last 12 months in Uganda. Analysis across regions (Table 3), indicates significance (moderate) in only central region, with respondents in central region who had one sexual partner were 0.55 times (OR = 0.55; CI = 0.31-0.98) less likely to have never tested for HIV compared with the sexually active men (15 – 54 years) who reported not to have had a sexual partner in the last 12 months.

## Discussion

The present study aimed at understanding the factors associated with never having tested for HIV among sexually active men (men who have had sexual intercourse in the last 12 months) aged 15 – 54 across regions of Uganda. Across regions of the country, about 20% of sexually active men aged 15 – 54 have never been tested for HIV. Findings established that in Uganda, age (25 – 34 years vs 15 – 24 years), education level (primary, secondary, and higher vs no education), religious status (muslim vs catholics), wealth quintile (richest vs poorest), worked in the last 12 months, marital status, circumcised, and number of sexual partners in the last 12 months (1 sexual partner vs no sexual partner), were associated with never to have tested for HIV among respondents; with analysis across regions of Uganda demonstrating amidst variations that, education level and marital status were the only factors associated with never to have tested for HIV among sexually active men (15 – 54 years). Conspicuously, age, religious status, wealth quintile, worked in the last 12 months, circumcised, and number of sexual partners in the last 12 months (excluding their spouses) were associated with never to have tested for HIV among respondents in particular regions.

Specifically, we found that amid variations in effect, an increase in education level (primary, secondary, and higher vs no education) was associated with reduced odds of never to have tested for HIV among respondents across regions. Our study agrees with findings in Uganda, and elsewhere that found increase in education level associated with HIV testing status [12, 32, 49, 63, 70, 71]. The most conceivable reason for this association could be that possessing secondary or higher education level can influence an individual’s desire for health, knowledge on access and uptake of health care services, including HIV testing services [71–78]. Consequently, the study finding stresses the need to promote men with no education to attain at least secondary educational level as a pathway to promote HIV testing among sexually active men aged 15 – 54 across the four administrative regions.

In addition, we found that never-married respondents were more likely not to have ever tested for HIV compared with the ever-married respondents, amid very strong significance across regions, except for eastern region that had a strong significance. There are limited studies done in Uganda that justify this significance; our study finding is in agreement with some studies in Uganda, and elsewhere [12, 32, 79–82]. The possible explanation for this association could be linked to the influence of the spouse, and or fathering of children which triggers behavioral change that is skewed towards improving health care seeking and service uptake, and cultural reforms [47, 55, 75, 83, 84]. Therefore, further examination should be done to understand the influence of marital status on HIV non-testing among respondents across the four administrative regions of Uganda.

Our study established that respondents in the richest wealth quintile (poorer, middle, richer, richest vs poorest) amidst very strong significance predicted HIV never testing among respondents. However, an analysis across regions demonstrated that wealth quintile was a major influence of HIV never testing only among respondents in western region, with moderate significance among respondents in the richest wealth quintile. There are limited studies that justify this difference in association and significance in central and western regions of Uganda. Available literature demonstrates that men who reported to have never tested for HIV belonged to the lower wealth quintile [12, 63], different from their counterparts in wealthier quintile [49, 70, 85]. The most plausible explanation for this association could be that less affluent men are taken-up most by economic opportunities that assist them meet the needs of their families [86]. Nevertheless, more research should be directed towards understanding this association in Central and Western region.

Furthermore, findings in the present study demonstrate that circumcised sexually active men (15 – 54 years) in central and western region amidst variations were less likely to have never been tested for HIV. Conversely, although a study in central region of Uganda and elsewhere in sub-Saharan Africa demonstrate that unlike HIV never tested men, ever tested men were more likely to be circumcised men [12, 32]. Limited studies explain this result, which therefore prompts further examination. However, it could be attributed to better health infrastructure in central and western regions [22], and medical male circumcision which is a means of preventing HIV contraction among men and its capacity to decrease the risk of HIV transmission by 60% [7]. Notably, before the procedure of medical male circumcision, a person is first supposed to test in order to obtain sero-status results that guide safety measures undertaken in the surgical procedure [7].

On the other hand, data used in this present study had some limitations. Firstly, the data was basically restricted to only variables captured in the 2016 UDHS. Therefore, an interrogation of crucial variables was limited, for instance; the data excluded respondents above the age of 54. Lastly, the cross-sectional kind of data used in the present study frustrated the capability to scrutinize casual relationships.

## Conclusions

Across regions of Uganda, about 20% of respondents never tested for HIV. Socio-economic and demographic factors associated with never having tested for HIV among sexually active men aged 15 – 54 across regions were; educational level and marital status. Age, religious status, wealth quintile, worked in the last 12 months, circumcised, number of sexual partners in the last 12 months (excluding spouses) were only correlates of never having tested for HIV among respondents in particular regions of the country. Therefore, deliberate efforts by the government of Uganda and relevant stakeholders need to be channeled towards the promotion of male education, and further examination into the association of HIV never testing among sexually active men (15 – 54 years) and being married across regions of the country. Also, efforts should be geared towards addressing regional variations in order to maximize HIV testing among sexually active men (15 – 54 years) across regions of Uganda, as well reduce on HIV related morbidity and mortality.

## Data Availability

The dataset that was used in the present study is available on rational request from the corresponding author. Authorization would as well be required from MEASURE DHS.

## Abbreviations

HIV: Human Immunodeficiency Virus
UDHS: Uganda Demographic and Health Survey
AIDS: Acquired immunodeficiency Syndrome
MSM: Men who have Sex with Men
UNAIDS: United Nations Programme on HIV/AIDS
WHO: World Health Organization
